# Europium Ion-Based Magnetic-Trapping and Fluorescence-Sensing Method for Detection of Pathogenic Bacteria

**DOI:** 10.1021/acs.analchem.4c00655

**Published:** 2024-03-25

**Authors:** Miftakhul Jannatin, Tzu-Ling Yang, Yi-Yuan Su, Ru-Tsun Mai, Yu-Chie Chen

**Affiliations:** † Department of Applied Chemistry, National Yang Ming Chiao Tung University, Hsinchu 300, Taiwan; ‡ Department of Biological Science and Technology, National Yang Ming Chiao Tung University, Hsinchu 300, Taiwan; § International College of Semiconductor Technology, National Yang Ming Chiao Tung University, Hsinchu 300, Taiwan

## Abstract

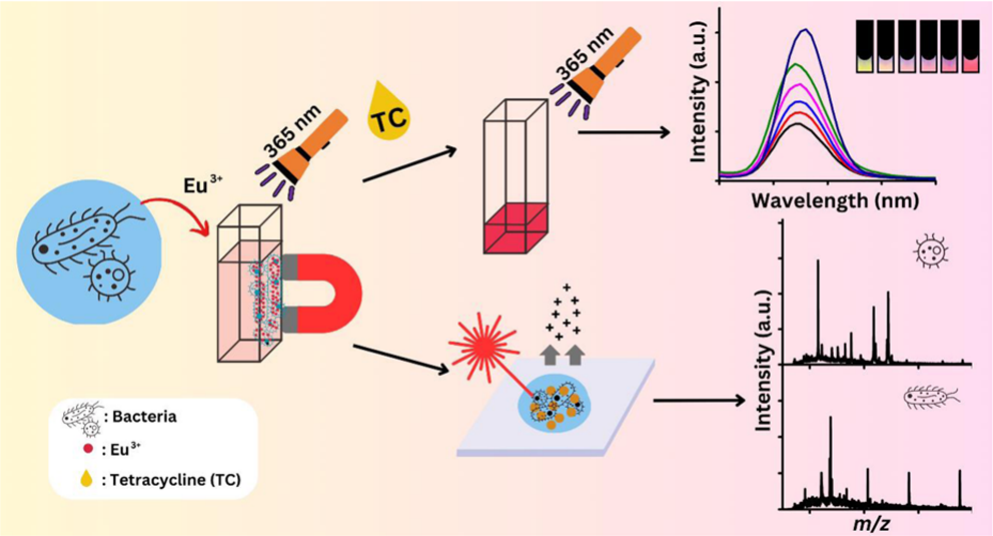

Europium ions (Eu^3+^) have been utilized as a fluorescence-sensing probe for a variety of analytes, including tetracycline (TC). When Eu^3+^ is chelated with TC, its fluorescence can be greatly enhanced. Moreover, Eu^3+^ possesses 6 unpaired electrons in its f orbital, which makes it paramagnetic. Being a hard acid, Eu^3+^ can chelate with hard bases, such as oxygen-containing functional groups (e.g., phosphates and carboxylates), present on the cell surface of pathogenic bacteria. Due to these properties, in this study, Eu^3+^ was explored as a magnetic-trapping and sensing probe against pathogenic bacteria present in complex samples. Eu^3+^ was used as a magnetic probe to trap bacteria such as *Staphylococcus aureus*, *Escherichia coli*, *Enterococcus faecalis*, *Acinetobacter baumannii*, *Bacillus cereus*, and *Pseudomonas aeruginosa*. The addition of TC facilitated the easy detection of magnetic Eu^3+^–bacterium conjugates through fluorescence spectroscopy, with a detection limit of approximately ∼10^4^ CFU mL^–1^. Additionally, matrix-assisted laser desorption/ionization mass spectrometry was employed to differentiate bacteria tapped by our magnetic probes.

## Introduction

Diseases caused by pathogens such as foodborne pathogenic bacteria have been a major threat to humans.^1^ Many people die or become ill due to infections caused by pathogenic bacteria.^2^*Staphylococcus aureus*, *Escherichia coli*, *Enterococcus faecalis*, *Acinetobacter baumannii*, *Bacillus cereus*, and *Pseudomonas aeruginosa* are common foodborne pathogens.^1,3,4^ Generally, these pathogens exist in complex samples.^5^ Classical methods with time-consuming overnight culture followed by bioassays are usually employed.^6^ Aptamers^7^ and antibody-based methods^8^ have been commonly used in bacterial identification due to their sensitivity and specificity. thods are cost-intensive and require sophisticated instruments,^9^ professional kits,^9^ or a long analysis time.^10^ Thus, to develop analytical methods that can be used to rapidly isolate and detect the presence of pathogenic bacteria from complex samples is important to prevent outbreaks caused by foodborne pathogens.^6^

Magnetic nanoparticles (MNPs) are useful probes for the enrichment and isolation of target bacteria from complex samples due to their magnetic property, leading to the elimination of the requirement of time-consuming overnight bacterial culture.^11−17^ Iron oxide MNPs immobilized with different metal oxides,^13,18,19^ polymers,^20^ aptamers,^21^ antibodies,^22^ proteins,^17^ glucose,^23^ peptides,^16^ and antibiotics^14^ have been used as affinity probes to target bacteria. Generation of such probes was usually time-consuming. Thus, a simple and rapid approach for magnetic isolation of bacteria from sample solution has been demonstrated.^24,25^ That is, magnetic metal ions, including Fe^3+^,^24^ Co^2+^,^24^ Ni^2+^,^24^ and Gd^3+ ^^25^ have been shown to effectively confer magnetism to both Gram-positive and Gram-negative bacteria by anchoring on the bacterial surface. This interaction occurs due to the presence of oxygen-containing functional groups such as phosphates, which act as hard Lewis bases, on the bacterial surface, and the above-mentioned magnetic metal ions, acting as either borderline or hard Lewis acids, according to the Hard Soft Acid Base (HSAB) theory.^26^ The resulting magnetic metal ion–bacteria conjugates have a high density of magnetic metal ions in a small space, i.e., a bacterial cell, and can be easily isolated by simply placing an external magnet.^24,25^

Eu^3+^ has been utilized as a biosensing probe for various target analytes due to its fluorescence capabilities.^27−30^ In addition to its fluorescence property, Eu^3+^ also exhibits paramagnetic properties owing to its electron configuration ([Xe]4f^6^), which makes it a potential candidate for use as a magnetic probe. That is, Eu^3+^ is a hard acid^31^ and has a high affinity to interact with oxygen-containing functional groups such as phosphates, according to the HSAB theory.^26^ The magnetism arises from the concentrated accumulation of magnetic metal ions within a confined area. Consequently, the resultant magnetic conjugates can be effectively separated by placing an external magnet (e.g., ∼4000 G).^24,25^ Therefore, micrometer-sized bacteria, which allow hard acids such as Eu^3+^^31^ to anchor and aggregate on their surface, are ideal targets for Eu^3+^. Typically, the fluorescence of Eu^3+^ can be enhanced by its chelating agents, such as TC.^32^ TC can chelate with Eu^3+^ over its tricarbonyl or β-diketone ring to form a complex.^33^ In the Eu^3+^–TC complex, TC absorbs the excitation light and transfers the energy to Eu^3+^ via the antenna effect,^32−34^ resulting in enhanced fluorescence emission from Eu^3+^. However, to date, no studies have reported on the utilization of Eu^3+^ as a magnetic probe to confer magnetism to nonmagnetic species such as bacteria. Therefore, in this study, we harnessed the unique properties of Eu^3+^, namely, fluorescence and magnetism, to develop a rapid sensing method for detecting bacteria. Namely, Eu^3+^ was used not only as a fluorescence-sensing probe but also as a magnetic probe. Moreover, mass spectrometry (MS), such as matrix-assisted laser desorption/ionization (MALDI)-MS, has been used to identify microorganisms based on the fingerprint mass spectra derived from intact microorganism cells.^17,18,35,36^ Thus, to differentiate bacteria trapped by Eu^3+^, MALDI-MS was used as a detection tool.

## Experimental Section

### Materials and Reagents

Europium(III) acetate (99.9%) was purchased from Alfa Aesar (Boston, MT). α-Cyano-4-hydroxycinnamic acid (CHCA), hydrogen peroxide, phosphoric acid, TC, tetramethyl benzidine (TMB), and sodium hydroxide were purchased from Sigma-Aldrich (St. Louis, MO). Hydrochloric acid (36.5%) and tris(hydroxymethyl)aminomethane (Tris) were obtained from J. T. Baker (Phillipsburg, NJ). Trifluoroacetic acid (TFA) (99%) was obtained from Riedel-de-Haen (Buchs, St. Gallen, Switzerland), whereas acetonitrile (99%) was obtained from Merck (Darmstadt, Germany). Yeast extract was obtained from Apolo Biochemical (Hsinchu, Taiwan). Tryptic soy broth (TSB) and Luria–Bertani (LB) were obtained from Neogen Culture Media (Taiwan). Sodium phosphate monobasic was purchased from Mallinckrodt Baker (Paris, KY). Pure water used in all experiments was purchased from Taisun (Taiwan). *S. aureus, P. aeruginosa*, and *E. faecalis* were collected from patients in the Hualien Tzu-Chi Hospital and provided by Prof. P.-J. Tsai (National Cheng-Kung University, Taiwan). *A. baumannii* M3237 was collected from a patient in the Hualien Tzu-Chi Hospital and provided by Prof. N.-T. (Tzu-Chi University, Taiwan). *E. coli* J96 was a gift from Dr. J. Johnson (Minneapolis Veterans Affairs Medical Center and University of Minnesota). *B. cereus* (BCRC 17427) was purchased from the Bioresource Collection and Research Center (Hsinchu, Taiwan). Neodymium magnets (∼4000 G), apple juice, and bleach were obtained from local shops.

### Instrumentation

Biochrom WPA C08000 cell density meter from BioPioneer (Cambridge, United Kingdom) was used to determine bacterial concentrations based on the optical density at a wavelength of 600 nm (OD_600_). A domestic microwave oven from Sampo (Taipei, Taiwan) was used to assist in the binding between Eu^3+^ and bacteria. All of the fluorescence spectra were obtained using a Jobin Yvon Horiba FluoroMax-3 spectrofluorometer (New Jersey). Mass spectra were obtained using a Bruker Daltonics Autoflex III MALDI time-of-flight mass spectrometer (Bremen, Germany) equipped with a solid laser (λ = 355 nm). Linear mode was used for acquiring the mass spectra of bacteria. The SEM images of bacteria were obtained using a JSM 7401F field-emission scanning electron microscope (SEM) (JEOL, Japan).

### Preparation of Bacterial Samples

Gram-positive bacteria, including *S. aureus, E. faecalis*, and *B. cereus*, were cultured in the agar plate containing agar and the mixture of TSB and yeast (TSBY). Gram-negative bacteria, including *P. aeruginosa, E. coli* J96, and *A. baumannii*, were cultured in the agar plate containing LB. All of the bacteria were incubated in an incubator at 37 °C for 12 h. Supporting Information (SI) Table S1 shows the list of the conversion of OD_600_ of 1 to the concentration of the model bacteria in terms of the unit of mg mL^–1^ and CFU mL^–1^. The resulting bacteria were rinsed with deionized water twice, followed by heating in a water bath at 60 °C for 30 min. The resultant bacteria were lyophilized and stored in a freezer (−20 °C) before use.

### Examination of the Optimal pH When Interacting Eu^3+^ with TC

TC was used as an enhancer to improve the fluorescence intensity derived from Eu^3+^. To find the optimal pH for binding TC with Eu^3+^, aqueous Eu^3+^ (2 mM) mixed with TC (13 μM) prepared in the buffers at different pH values were incubated under vortex-mixing for 1 h. The optimal experimental parameters were determined according to the fluorescence spectral results derived from Eu^3+^ (λ_ex_ = 365 nm).

### Examination of the Optimal Parameters for Binding Eu^3+^ onto Bacteria

*S. aureus* was selected as the model bacterium. Aqueous europium(III) acetate (0.4 M, 10 μL) was added to the samples (0.39 mL) containing *S. aureus* (0.2 mg L^–1^) prepared in the buffers at different pH values. Ammonium acetate (10 mM) was used to prepare buffer at pH 6, whereas Tris (10 mM) was used to prepare buffers at pH 7, 8, and 9. Each bacterial sample added with Eu^3+^ was placed in a water bath (2.5 mL), followed by being subjected to a microwave oven (power: 180 W) for 1–2.25 min or vortex-mixing for 0.5–1 h. After cooling to room temperature, the Eu^3+^-bacterium conjugates in the glass vial (maximum volume: ∼0.45 mL; wall thickness: ∼0.5 mm) were magnetically isolated by placing an external magnet (∼4000 G) for 30 min. The supernatant (0.37 mL) was removed, and the remaining conjugates were rinsed with the buffer.

### Examination of the Optimal Concentration of Bleach Added to the Bacterium–Eu^3+^ Conjugates

To release Eu^3+^ from the suspension of the bacterium–Eu^3+^ conjugates, bleach (10 μL) with different concentrations (0.5, 0.25, and 0.2%) was added to the bacterium–Eu^3+^ conjugates above and incubated under microwave heating (power: 180 W) for 1.75 min. That is, the final concentrations of bleach in the samples were 0.125, 0.0625, and 0.05%. The resulting sample was added with TC (30 μM, 30 μL) and incubated under microwave heating (power: 180 W) for 1.75 min, followed by analysis by fluorescence spectroscopy to determine the optimal concentration of bleach added in the bacterium–Eu^3+^ conjugates.

### Using Eu^3+^ as the Trapping and Sensing Probe against Bacteria

**Scheme 1:**
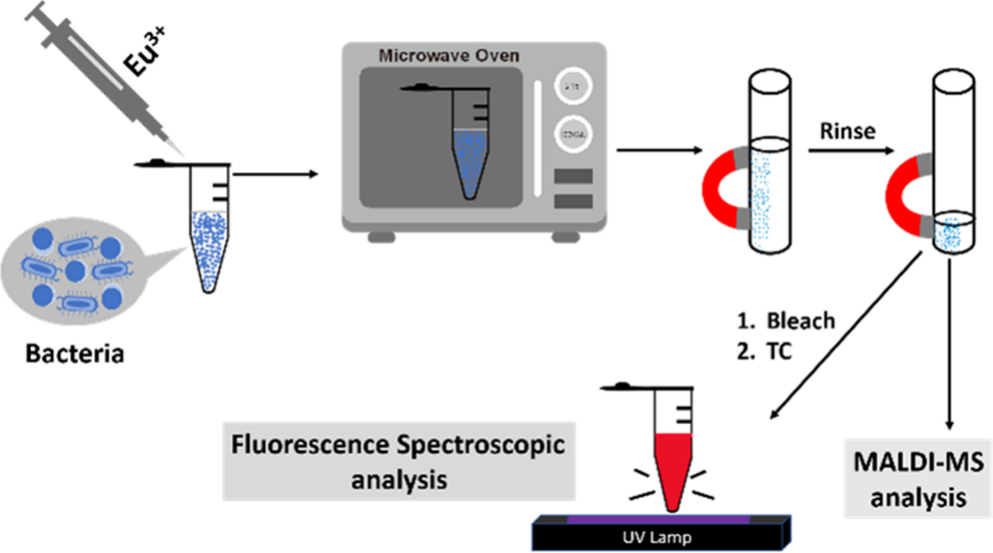
Cartoon Illustration of the Experimental Steps of Our Method

Scheme 1 shows a cartoon illustration of our approach using Eu^3+^ as the trapping and sensing probes toward bacteria. Model bacteria with different concentrations were prepared in Tris buffer (pH 8). Aqueous europium(III) acetate (75 mM, 10 μL) was added to the sample (0.39 mL) containing model bacteria at pH 8 and incubated in a microwave oven (power: 180 W) for 2.25 min. The conjugates were then magnetically isolated by placing an external magnet (∼4000 G), followed by rinsing with the same buffer (0.4 mL × 2) and elimination of the rinse buffer. The volume of the resulting suspension was ∼30 μL. Bleach (0.25%, 10 μL) prepared in Tris buffer (pH 8) was added to the suspension above and incubated in a microwave oven (power: 180 W) for 1.75 min. The resulting mixture was added with TC (30 nM, 30 μL) prepared in Tris buffer at pH 8.5 and incubated in a microwave oven (power: 180 W) for another 1.75 min. The resulting sample was examined by fluorescence spectroscopy (λ_ex_ = 364 nm).

### Examination of the Effects of Interference Species

Metal ions (K^+^, Na^+^, Mg^2+^, and Ca^2+^), saccharides (glucose and sucrose), and acids (malic and ascorbic acids) were selected as the interference species. Tris buffer (10 mM, pH 8) containing these interference species was used to prepare bacterial samples. The concentrations of Mg^2+^, K^+^, Ca^2+^, Na^+^, malic acid, ascorbic acid, sucrose, and glucose were 0.10, 2.02, 0.16, 0.08, 0.90, 0.02, 25.2, and 52.6 mg mL^–1^, respectively. The concentrations of the interference species were determined based on the typical concentrations of these species in real-world samples that have been suggested by the United States FoodData Central.^37^*B. cereus* with the concentration of 0.02 mg mL^–1^ was spiked in the as-prepared samples containing those interference species above. The trapping and sensing steps were the same as those described in the section above.

### Analysis of the Simulated Real Samples

A 500-fold diluted apple juice prepared in Tris buffer (10 mM, pH 8) was spiked with *B. cereus* (500 ng mL^–1^), which was used as the simulated real sample for evaluation of the quantitative analysis using the developed method. The standard addition method was used to determine the concentration of *B. cereus* in the apple juice sample prepared as described above. That is, additional *B. cereus* with different concentrations (50–800 ng mL^–1^) were spiked into the simulated real samples. The rest of the experimental steps using Eu^3+^ as the trapping and sensing probes were the same as stated above.

### Using MALDI-MS for Bacterial Characterization

The sample preparation and the trapping steps for the bacterial samples were similar to what was stated above when using MALDI-MS as the detection tool. *S. aureus, E. faecalis*, and *A. baumannii*, with the concentration of 0.8 μg mL^–1^ were used as the model samples. After magnetic isolation using Eu^3+^ as the probe, the resulting conjugates were mixed with CHCA (20 mg mL^–1^, 1.5 μL) prepared in the solvent containing acetonitrile and aqueous TFA (3%) (v/v, 2:1). After solvent evaporation, the samples were introduced into the MALDI mass spectrometer for MS analysis.

## Results and Discussion

### Using Eu^3+^ as the Magnetic Probe for Trapping Bacteria

**Figure 1:**
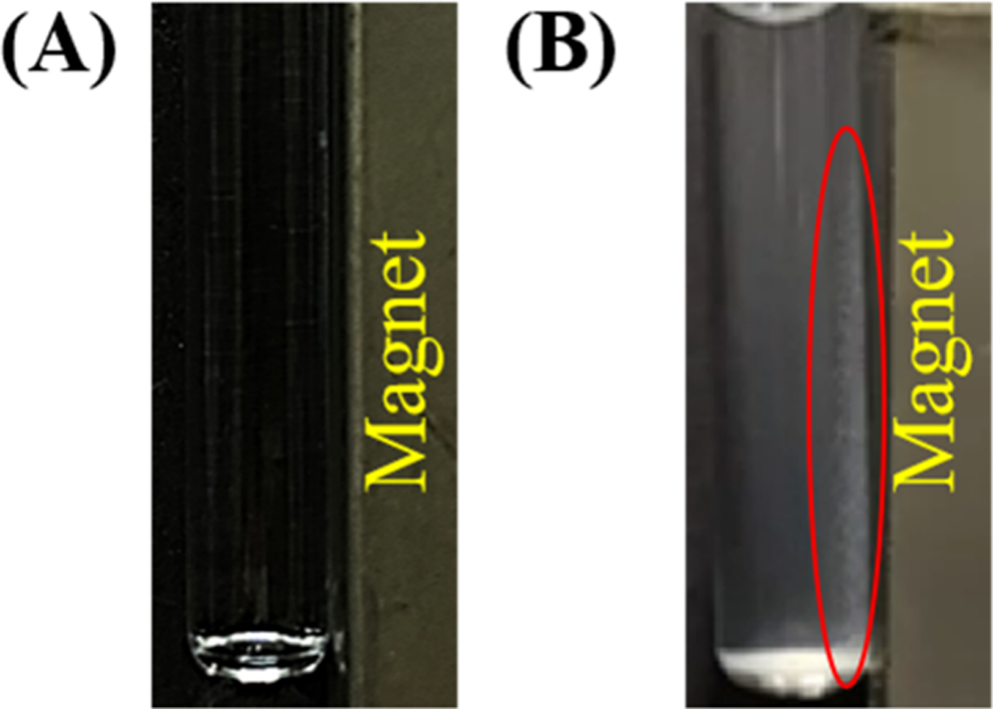
Examination of the magnetic Eu^3+^–bacterium conjugates. Photographs of the (A) blank sample (0.39 mL) containing Tris buffer at pH 8 only and (B) the bacterial samples (0.39 mL, 0.5 mg mL^–1^) containing *S. aureus* prepared in Tris buffer (pH 8) obtained with the addition of Eu^3+^ (75 mM, 10 μL), followed by microwave heating (power: 180 W) for 2.25 min and magnetic isolation by placing an external neodymium magnet (∼4000 G). The photographs were taken under room light. The red oval indicates the location of the magnetic Eu^3+^–bacterium conjugates.

Gram-positive bacteria, including *S. aureus*, *E. faecalis*, and *B. cereus*, and Gram-negative bacteria, including *P. aeruginosa, E. coli* J96, and *A. baumannii*, were initially used as the model samples to examine whether Eu^3+^ can be used as the magnetic-trapping probe. Figure 1A shows the photograph of Eu^3+^ alone prepared in Tris buffer, whereas Figure 1B shows the photograph of *S. aureus* trapped by Eu^3+^, followed by magnetic isolation. A magnet was placed on the right side of each sample vial. No precipitates or magnetic conjugates derived from the sample containing Eu^3+^ alone were observed (Figure 1A and Supporting Information time-lapse Video 1). Apparent white spots (circled in red) containing the Eu^3+^–bacterium conjugates were adhered to the wall of the glass vials next to the magnet (Figure 1B and Supporting Information time-lapse Video 2). Similar phenomena were observed from the samples containing other model bacteria that were mixed with Eu^3+^ (Supporting Information Figure S1). These results indicated that Eu^3+^ could be used as a trapping probe to magnetically isolate these model bacteria. Moreover, the Eu^3+^–bacterium conjugates exhibited a substantial increase in magnetism, rendering them highly susceptible to aggregation upon placement of an external magnet. Additionally, Tris was chosen as the buffer in this study due to the tendency of hard acids, such as Eu^3+^, to readily interact with hard bases like phosphates,^31^ which are present in phosphate-buffered saline (PBS). Therefore, buffers containing hard bases should be avoided when implementing the developed method.

Moreover, given that microwave heating was employed to accelerate the binding of Eu^3+^ to bacterial cells, we investigated whether the bacterial cells could maintain their integrity. Supporting Information Figure S2A,B displays the SEM images of *S. aureus* obtained before and after microwave heating. Evidently, the morphology of the bacterial cells remained unchanged after microwave heating.

### Using Eu^3+^ as the Fluorescence-Sensing Probe

TC has been considered as an effective fluorescence enhancer for Eu^3+^.^32−34^ The optimal pH for enhancing the fluorescence derived from Eu^3+^ with the addition of TC was examined initially (SI Figure S3A–S3F). SI Figure S3G shows the bar graphs of the summarized results from Figure S3A–S3F. Apparently, pH 8.5 worked the best. However, the fluorescence intensity dropped at pH 9. Presumably, more hydroxyl ions existed in the sample at pH 9 than at lower pH values. They competed for the binding between Eu^3+^ and TC, resulting in the decrease of the fluorescence enhancement of Eu^3+^ assisted by TC.^38,39^ Thus, when TC was used to enhance the fluorescence intensity derived from Eu^3+^ attached on target bacteria, the pH value of the buffer was adjusted to 8.5 in the following studies.

After finding the optimal pH, we further used TC to enhance the fluorescence intensity derived from Eu^3+^ on bacteria. Given that the fluorescence of Eu^3+^ can be enhanced when chelating with TC, we decomposed bacteria to release Eu^3+^ from the Eu^3+^–bacterium conjugates using bleach, which contained abundant OCl^–^ and could destroy the bacterial cells. SI Figure S4 shows the fluorescence spectra of the sample containing *S. aureus* mixed with Eu^3+^ obtained after being trapped by Eu^3+^, followed by bleach treatment and the addition of TC. Apparently, 6.25 × 10^–2^% bleach worked the best and was used to treat the bacteria trapped by Eu^3+^ prior to the addition of TC in the following studies.

In addition, the optimal incubation condition of interacting TC with the Eu^3+^–bacterium conjugates after treatment with bleach was examined. SI Figure S5 shows the resultant fluorescence spectra of the samples obtained under different incubation conditions. Apparently, the sample incubated under microwave heating (power: 180 W) for 1.75 min showed the highest fluorescence intensity (green), which was even higher than that was incubated under vortex-mixing for 2 h (red). Thus, microwave heating for 1.75 min (power: 180 W) was used as the incubation condition when TC was interacting with the Eu^3+^–bacterium conjugates that had been treated by bleach.

SI Figure S6 shows the fluorescence spectra of the samples containing *S. aureus, P. aeruginosa*, *E. faecalis, B. cereus, E. coli* J96, *A. baumannii*, and blank obtained using Eu^3+^ as the trapping and sensing probe with the optimized experimental parameters obtained above. The fluorescence intensity of the bacterial samples (blue spectra) was significantly increased, obtained after treatment with bleach, followed by the addition of TC. These results (Figure 1 and SI Figure S6) indicated that Eu^3+^ can play two roles as magnetic-trapping and fluorescence-sensing probes against these model bacteria.

### Optimization of Experimental Parameters

The optimal experimental parameters, including pH and the incubation time for Eu^3+^ to bind with bacteria, were examined. *S. aureus* was used as a model bacterium. SI Figure S7A–S7E shows the representative fluorescence spectra of the supernatants of the samples containing Eu^3+^ prepared at different pH values obtained before (black) and after (blue) being incubated with *S. aureus* followed by centrifugation. The intensity difference at the wavelength of 616 nm between the black (I_0_) and blue (I_i_) bands indicated the binding capacity of Eu^3+^ on *S. aureus* obtained before and after (blue), respectively, incubated with *S. aureus*. SI Figure S7F shows the summarized results of the binding capacity of *S. aureus* toward Eu^3+^ obtained from different pH values from three replicates. The maximum binding capacity of *S. aureus* toward Eu^3+^, occurs at pH 8. Namely, the results indicated that the optimal pH for Eu^3+^ to bind onto *S. aureus* was pH 8. Tris buffer was used because other buffers such as phosphates in PBS buffer can easily interact with Eu^3+^ and cause obstacles when trapping target bacteria. Such buffers should be avoided.

In addition, the optimal incubation conditions were also examined. SI Figure S8A–S8G shows the representative fluorescence spectra of the supernatants of the samples containing Eu^3+^ without (black) and with (blue) the addition of *S. aureus* under different incubation conditions, followed by centrifugation. In addition to vortex-mixing, microwave heating, which has been known to be useful in the acceleration of binding processes^40^ was examined to see whether the incubation time could be shortened. SI Figure S8H shows the summarized results from three replicates obtained under different incubation conditions. Apparently, the binding capacity of Eu^3+^ on *S. aureus* obtained under microwave heating (power: 180 W) for 2.25 min was the highest. Thus, pH 8 and incubation time under microwave heating (180 W) for 2.25 min were used for binding Eu^3+^ onto bacteria in the following studies.

### Examination of Quantitative Analysis and Limit of Detection

We further examined whether our method could be suitable for quantitative analysis. *S. aureus* was selected as the model bacteria. SI Figure S9 shows the resulting fluorescence spectra of *S. aureus* with different concentrations. SI Figure S10 shows the resulting plots by plotting the fluorescence intensity at 616 nm obtained from SI Figure S9 versus the concentration of *S. aureus*. According to the obtained equation (*y* = 5.392 × 10^–1^*x* + 3.150 × 10^5^, *R*^2^ = 0.9972) listed in the inset of SI Figure S10, the limited of detection (LOD) for the model bacteria was estimated to be ∼3.5 × 10^4^ CFU mL^–1^ based on 3δ/S, in which δ and S stand for the standard deviation of the intercept (i.e., 6294) on *Y* axis and the slope (i.e., 0.5392) of the calibration curve, respectively.

### Examination of the Effects of Interference Species

**Figure 2:**
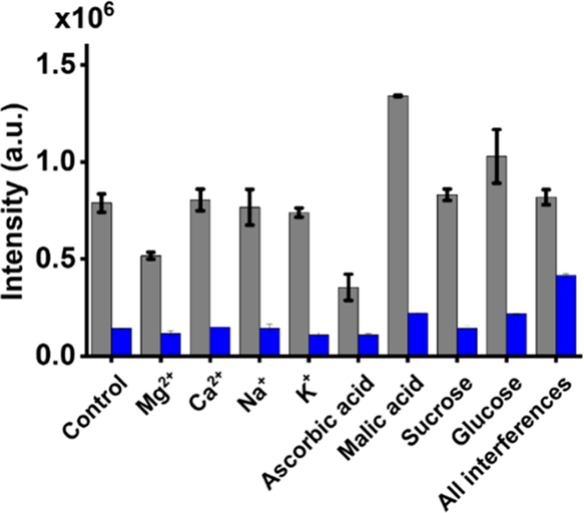
Examination of the effects of interference species. Bar graphs showing the summarized results (SI Figure S9) of the fluorescence intensity at 616 nm of the fluorescence spectra of the samples containing *B. cereus* (0.2 mg mL^–1^) and interference species (gray) and interference species alone (blue) prepared in Tris buffer (pH 8) obtained by adding Eu^3+^ (75 mM, 10 μL) as the trapping and sensing probes, followed by bleach treatment and the addition of TC (30 μM, 30 μL).

We further examined whether interference species that commonly exist in real-world samples may affect our sensing results. Potassium ions, sodium ions, magnesium ions, calcium ions, glucose, sucrose, malic acid, and ascorbic acid that are commonly found in drinks such as juice^37^ were selected as the interference species. *B. cereus* was used as the model sample. SI Figures S11A–S11P show the resulting fluorescence spectra. Figure 2 shows the bar graphs (gray) of the summarized results of the samples containing *B. cereus* (2 μg mL^–1^) in the presence of interference species obtained after using our trapping and sensing method. The blue bar graphs show the results obtained from the samples containing interference species alone. The details of the experimental steps are described in the Experimental section. The gray and green bar graphs labeled with the control were obtained from *B. cereus* prepared in Tris buffer (pH 8) and Tris buffer only, respectively. Apparently, the intensity resulting from the samples containing *B. cereus* was higher than those not containing *B. cereus.* The resulting fluorescence intensity obtained from the bacterial samples containing the interference species, including Mg^2+^ and ascorbic acid, was apparently lower than that obtained from the control sample without the presence of any interference species. That is, the presence of these interference species affected the sensing results to a certain extent. Presumably, Mg^2+^ competed with Eu^3+^ to bind with *B. cereus*, and ascorbic acid competed with *B. cereus* to bind with Eu^3+^, leading to the reduction of the fluorescence derived from Eu^3+^. On the contrary, the presence of malic acid in the sample can enhance the fluorescence derived from Eu^3+^. It was because magnetic Eu^3+^–malic acid conjugates could be formed and isolated together with Eu^3+^–bacterium conjugates during magnetic isolation, resulting in the enhancement of the fluorescence intensity derived from Eu^3+^ after the addition of TC. These results indicated that the presence of certain interference species would affect the sensing results, although most of them did not cause significant effects. Nevertheless, the interference effects became very low when adding all of the interference species to the bacterial sample. The fluorescence intensity derived from the bacterial samples without and with the presence of all of the interferences appeared similar. This was understandable because some of the interference species enhanced the fluorescence, while others reduced the fluorescence derived from Eu^3+^. As a result, the overall effects became negligible.

### Examination of Precision and Accuracy

To examine the precision and accuracy of the developed method, we prepared a sample containing *S. aureus* with the concentration of ∼150 ng mL^–1^ as the sample and examined the same sample 6 times a day for 5 days using the developed method. The calibration curve (*y* = 5.392 × 10^–1^*x* + 3.15 × 10^5^, *R*^2^ = 0.997) shown in SI Figure S10 was used to determine the concentration of the sample from each run. SI Figure S12 shows the resulting fluorescence spectra, whereas SI Table S2 shows the summarized results. Accordingly, the precision and the accuracy were estimated to be 12.2 and 99.1%, respectively. These results demonstrated that using our method can obtain desirable results with good reproducibility and accuracy.

### Analysis of the Simulated Real Sample

**Figure 3:**
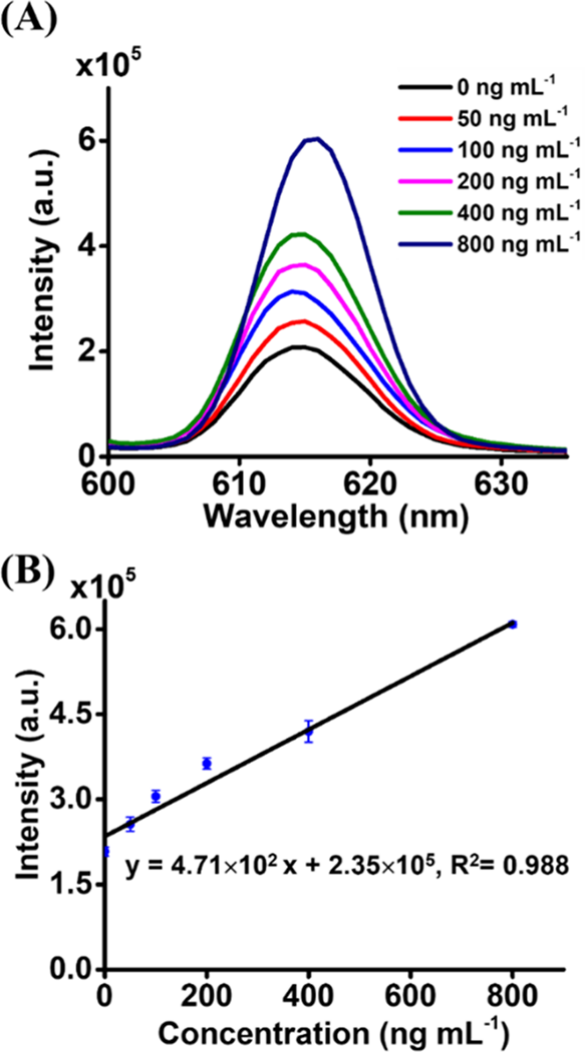
Quantitative analysis of *B. cereus* in an apple juice sample by using the standard addition method. (A) Representative fluorescence spectra of the apple juice sample containing *B. cereus* (∼500 ng mL^–1^) added with additional *B. cereus* at different concentrations obtained after using our trapping and sensing method. Three replicates were conducted. (B) Corresponding plot derived from the results in panel (A).

We further examined whether our method can be used to quantify bacteria in the simulated real sample. Apple juice spiked with *B. cereus* (500 ng mL^–1^) prepared in Tris buffer at pH 8 was used as the sample. Considering the matrix effect, the standard addition method was used to determine the concentration in the as-prepared real samples. *B. cereus* with five different concentrations was spiked in the as-prepared samples, followed by trapping and sensing using our method. Figure 3A shows the resultant fluorescence spectra, whereas Figure 3B shows the corresponding plot (*y* = 4.71 × 10^2^*x* + 2.35 × 10^5^, *R*^2^ = 0.988) by plotting the intensity at 616 nm of the resulting fluorescence spectra versus the concentration of *B. cereus* spiked in the samples. Accordingly, the concentration of *B. cereus* in the sample was estimated to be ∼499 ng mL^–1^, which was close to the spiked concentration, 500 ng mL^–1^.

### MALDI-MS Analysis

**Figure 4:**
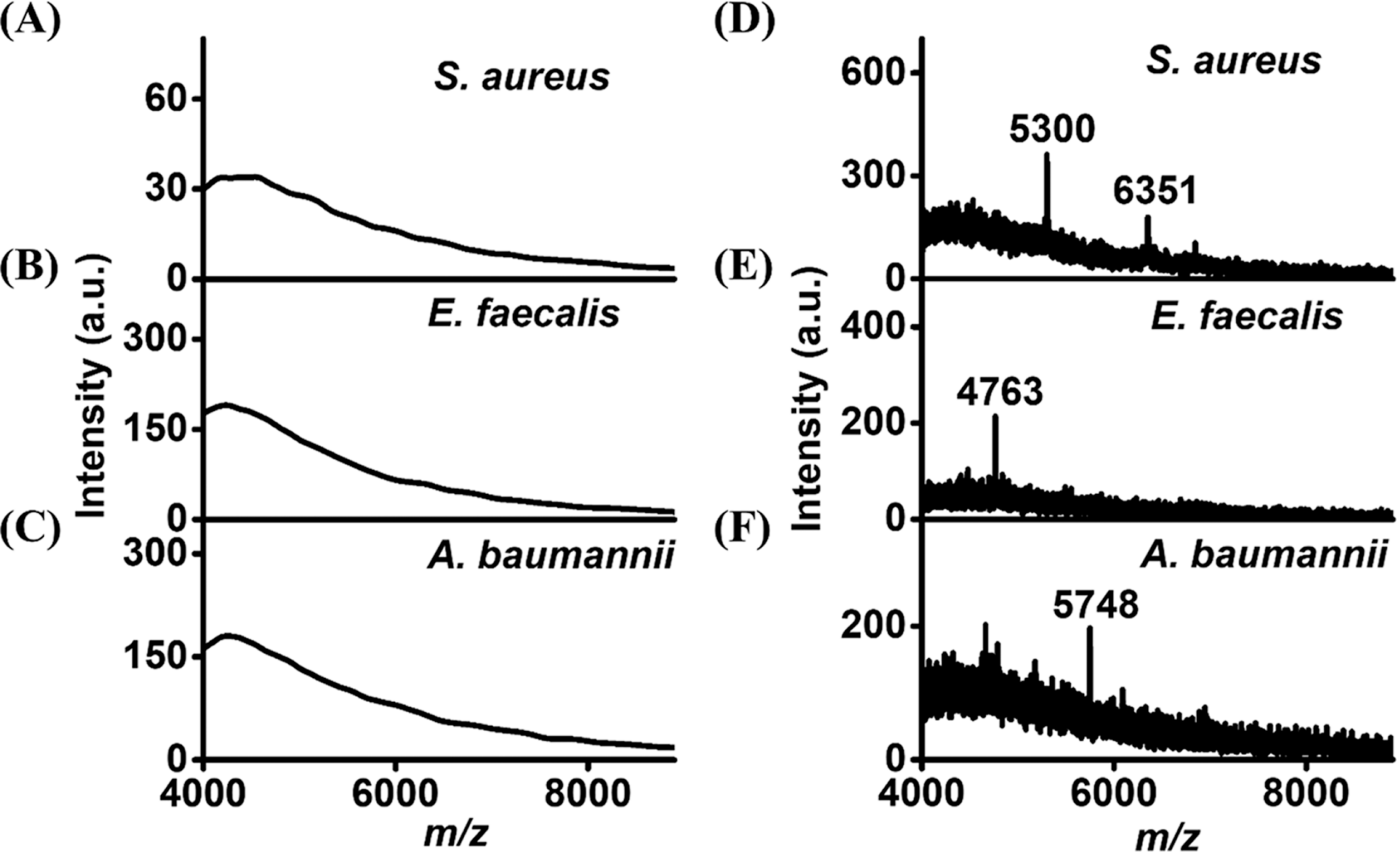
MALDI mass spectra of the samples (∼800 ng mL^–1^, 1.5 μL) containing (A) *S. aureus*, (B) *E. faecalis*, and (C) *A. baumannii* obtained before enrichment. (D–F) Corresponding mass spectra of the three bacterial samples (∼800 ng mL^–1^, 390 μL) obtained after enrichment by Eu^3+^. CHCA (20 mg mL^–1^) prepared in the solvent containing acetonitrile and 3% TFA (2:1, v/v) was used as the MALDI matrix.

Given that our fluorescence-sensing method cannot be used to distinguish different bacteria, we further employed MALDI-MS, which can be used to characterize bacteria based on their fingerprint mass spectra to identify bacteria that were trapped by Eu^3+^ via magnetic isolation. *S. aureus, E. faecalis*, and *A. baumannii* were selected as the model bacteria. SI Figure S13 shows the MALDI fingerprint mass spectra of these three model bacteria. Apparently, these three fingerprint mass spectra look quite different. Figure 4A–C shows the direct MALDI mass spectra of the bacterial samples, including *S. aureus, E. faecalis*, and *A. baumannii*, obtained before being concentrated by Eu^3+^. No apparent ions were observed in the resulting mass spectra owing to the low bacterial concentrations. Figure 4D–F shows the MALDI mass spectra of the same samples used to obtain Figure 4A–C obtained after enrichment by Eu^3+^. The peaks at *m*/*z* 5300 and 6351 in Figure 4D were the characteristic peaks standing for *S. aureus* (cf. Figure S13A). The peak at *m*/*z* 4763 in Figure 4E was derived from *E. faecalis* (cf. Figure S13B). Figure 4F was dominated by the peak at *m*/*z* of 5748, corresponding to the main peak derived from *A. baumannii* (cf. Figure S13C). These results indicated that MALDI-MS can be used to further identify the bacteria trapped by our magnetic probe.

### Comparison of Our Method with the Existing Methods

Supporting Information Table S3 lists the comparison of our method with the existing fluorescence-based sensing methods^41−45^ for detecting pathogenic bacteria. Evidently, the primary advantage of our approach lies in the short preparation time (∼2 min) of the sensing probes, specifically Eu^3+^. The probe can be easily prepared by dissolving europium acetate in an aqueous solution. In contrast, sensing probes produced by other existing methods required varying durations, ranging from a few tens of minutes to several tens of hours.^41−59^ As mentioned earlier, the LOD of our method for *S. aureus* was approximately 10^4^ CFU mL^–1^, determined through the calibration curve presented in Supporting Information Figure S10. When our LOD is compared with those obtained from other existing methods, it becomes apparent that our LOD is higher than those achieved by the existing methods. It is important to note, however, that the LODs determined by these existing methods were based on semilog graphs, where the logarithm of the concentration was plotted on the *X*-axis, as indicated in Supporting Information Table S3. Hence, the reported LODs were achieved using semilog graphs, although the criteria for employing this approach are not explicitly stated in the literature. We were able to determine the LOD of our method against *S. aureus* to be as low as ∼2 CFU mL^–1^ when utilizing a semilog graph as the calibration curve. Nevertheless, we are cautious about claiming the ability of our method to detect bacteria at such a low concentration level and are concerned about determining the LOD through such a graph.^60^ Therefore, we prefer to state that the LOD of our method is ∼10^4^ CFU mL^–1^. Moreover, the time required for the sensing experiments ranged from a few tens of minutes to a few hours. Unlike most methods that lack magnetic enrichment, our approach benefits from the use of Eu^3+^ as the magnetic-trapping probe, providing a distinct advantage.

## Conclusions

Rapid identification of the presence of pathogenic bacteria in complex samples is crucial for clarifying the sources of contamination and infection. By eliminating the time-consuming step of the overnight culture, our study successfully demonstrated that Eu^3+^ can serve a dual role as a trapping and sensing probe for bacteria. This marks the first report to utilize Eu^3+^ as a magnetic-trapping and fluorescence-sensing probe against bacteria. Affinity methods for enriching trace bacteria from complex samples are useful. The bacterium–Eu^3+^ conjugates can be easily isolated in a sample solution by using an external magnet. To enhance the fluorescence intensity, TC was added to the resulting samples, achieving a low limit of detection (∼10^4^ CFU mL^–1^) for *S. aureus*. The key strengths of the developed method are its simplicity and speed, making it a promising candidate for point-of-care applications. Its rapid screening capability opens up new possibilities for swiftly and efficiently identifying the presence of bacteria, which is vital in safeguarding public health and food safety, where bacterial contamination represents a significant threat. Furthermore, our approach can be expanded to applications beyond bacteria, including fungi and viruses. Supporting Information Figure S14 demonstrates the potential use of our method for sensing *Aspergillus niger* spores and lentiviruses. The color observed with *A. niger* spores and lentiviruses was evidently distinct from that observed in the blank samples without any microorganisms. However, additional efforts are needed to explore the optimal experimental conditions for assessing the sensitivity of our method to entities other than bacteria. Moreover, analytical tools, such as MALDI-MS, are required to further identify the species trapped in the magnetic conjugates.

## Supporting Information Available

Additional experimental details: preparation of fungal spores and lentiviruses, using Eu^3+^ as the magnetic-trapping and sensing probes for other microorganisms; conversion of OD_600_ of 1 with the weight and CFU per mL; examination of the accuracy and precision of the developed method; list of comparisons between existing methods and our work; examination of the magnetic Eu^3+^–bacterium conjugates; SEM images of *S. aureus;* examination of the optimal pH; optimization of the concentration of the added bleach; examination of incubation conditions; examination of different model bacteria; examination of the optimal binding pH of Eu^3+^ toward *S. aureus*; examination of the optimal incubation parameters; quantitative analysis; calibration curve; examination of interference effects; examination of precision and accuracy; characterization of fungal spores by MALDI-MS; examination of our method for detection of other microorganisms (PDF)
Time-lapse Video 1: Eu^3+^ in Tris buffer with an external magnet (MP4)
Time-lapse Video 2: *S. aureus* with Eu^3+^ in Tris buffer with an external magnet (MP4)
