# Supplementary material for: Europium Ion-Based Magnetic-Trapping and Fluorescence-Sensing Method for Detection of Pathogenic Bacteria
Source: Anal Chem. 2024 Mar 25;96(14):5669–76. doi: 10.1021/acs.analchem.4c00655 (PMC11007678; doi:10.1021/acs.analchem.4c00655)
Supplement: Supplementary file 1 — ac4c00655_si_001.pdf [file ac4c00655_si_001.pdf]

Supporting Information

Europium Ion-based Magnetic Trapping and Fluorescence-sensing Method for Detection of Pathogenic Bacteria

Miftakhul Jannatin,[1] Tzu-Ling Yang,[1] Yi-Yuan Su,[2] Ru-Tsun Mai,[2] Yu-Chie Chen[1,3*]

[1]Department of Applied Chemistry, National Yang Ming Chiao Tung University, Hsinchu 300, Taiwan

[2]Department of Biological Science and Technology, National Yang Ming Chiao Tung University, Hsinchu 300, Taiwan

[3]International College of Semiconductor Technology, National Yang Ming Chiao Tung University, Hsinchu 300, Taiwan

[*]Corresponding author

yuchie@nycu.edu.tw

Table of Contents

Additional experimental details S2

Table S1. Conversion of OD_{600} of 1 with the weight and CFU per mL S3

Table S2. Examination of the accuracy and precision of the developed method S3

Table S3. List of comparisons between existing methods and our work........ S4

Figure S1. Examination of the magnetic Eu^{3+}-bacterium conjugates. S6

Figure S2. SEM images of *S. aureus*........ S6

Figure S3. Examination of the optimal pH S7

Figure S4. Optimization of the concentration of the added bleach S7

Figure S5. Examination of incubation conditions S8

Figure S6. Examination of different model bacteria S9

Figure S7. Examination of the optimal binding pH of Eu^{3+} toward *S. aureus* S9

Figure S8. Examination of the optimal incubation parameters S10

Figure S9. Quantitative analysis S10

Figure S10. Calibration curve S11

Figure S11. Examination of interference effects S12

Figure S12. Examination of precision and accuracy........ S13

Figure S13. Characterization of fungal spores by MALDI-MS S13

Figure S14. Examination of our method for detection of other microorganisms S14

Additional Experimental Details

Preparation of fungal spores

Aspergillus niger (BCRC30130) was purchased from the Bioresource Collection and Research Center (Hsinchu, Taiwan) and grew in potato dextrose agar (HiMedia, Mumbai, India) at 28 °C for 7 days. The spores were collected by depositing with Tris buffer on the agar plate followed by picking up using a pipette.

Preparation of lentiviruses

Human embryonic kidney (HEK) 293 FT cells were grown in DMEM (#SH30003.02, Cytiva, Marlborough, MA, USA) supplemented with 10% heat-inactivated fetal bovine serum (#SH30396.13, Gibco, Grand Island, NY, USA), L-glutamine, nonessential amino acids (#CC517-0100, GeneDirectX, Taoyuan, Taiwan), and penicillin/streptomycin (#SV30010, Cytiva) in a humidified incubator (Forma 301, Thermo Fisher Scientific, Marietta, OH, USA) at 37°C under 5% CO_2. The plasmids pLKO.1-puro-based shLuc (TRCN0000072246), pCMV-ΔR8.74psPAX2 (expressing *pol*, *gag*, and *rev*), and pMD2.G (expressing vesicular stomatitis virus glycoprotein) were purchased from the RNAi Core Facility, Academia Sinica, Taiwan. For lentivirus production, HEK293FT cells were transfected with the plasmids described above using Lipofectamine 2000 transfection reagent (Invitrogen, Karlsruhe, Germany) according to the protocol suggested by the RNAi Core Facility (http://rnai.genmed.sinica.edu.tw/webContent/web/protocols).

Using Eu^{3+} as the magnetic trapping and sensing probes for other microorganisms

The trapping and sensing steps using Eu^{3+} as the probe were performed consistent with the description provided in the main text. However, in the case of magnetic aggregated fungal spores, 1.2% bleach (10 μL) was introduced instead of 0.25% bleach (10 μL). This was done because spores are inherently more resistant to dissolution than bacteria and viruses.

Table S1. Conversion of OD_{600} of 1 with the concentration and CFU mL^{-1} (n= 4)

Bacteria at OD_{600} of 1	weight (mg mL^{-1})	CFU mL^{-1}
S. aureus	0.50±0.04	~3.93×10^9
B. cereus	0.31±0.02	~3.76×10^9
E. faecalis	0.48±0.05	~1.19×10^8
E. coli J96	0.39±0.03	~1.75×10^9
A. baumannii M3237	0.57±0.03	~3.97×10^8
P. aeruginosa	0.69±0.06	~2.07×10^9

Table S2. Evaluation of the accuracy and the precision of the developed method.

	**Day 1**	**Day 2**	**Day 3**	**Day 4**	**Day 5**
Morning	175.9	165.9	147.9	123.8	177.0
	179.1	142.6	130.2	150.7	127.2
	174.7	137.6	127.1	161.2	127.3
Afternoon	164.1	167.3	142.2	164.2	127.5
	127.2	159.4	140.1	158.9	126.5
	174.7	152.6	160.2	153.3	173.5
Mean	165.9	154.2	141.3	152.0	143.2
% RSD	11.8	7.9	8.5	9.7	17.4
		Mean= 151.3±18.5			
		RSD% 12.2%		Accuracy	99.1%

Table S3. List of comparisons between existing methods and our work.

Fluorescence sensing probes[a]	Target bacteria	Time for synthesis of the probes	Semi-log plot: Y vs. log X	LOD (CFU mL^{-1})	Analysis time	Magnetic enrichment	Ref
Au NCs and $Ru(bpy)_3^{2+}$	*Staphylococcus aureus*	50 min	Yes	1.0	30 min	No	1
Apt-Van-QDs	*S. aureus*	> 8 h	Yes	1	30 min	No	2
NCND/GO/apt-o-CD	*Acinetobacter baumannii*	>88 h	Yes	100	20 min	No	3
Bi_2S_3@MNo$_2$@Vancomicyn	*S. aureus*	>18 h	Yes	6	10 min	No	4
BCD@SiO_2@BSA-AuNC	*Escherichia coli, Pseudomonas aeruginosa, and Salmonella typhimurium*	>75 h	Yes	150, 112, 792	>1.5 h	No	5
biocompatible CsPbBr3 perovskite quantum dots (BPQDs)	*E. coli*	>4.5 h	-	1.8	5 min	No	6
Aptamer-QDs-Teico-AuNPs	*S. aureus*	0.5 h	-	1	2 h	No	7
MTPE-FCyP/LUM/ZIF@FL	*E. coli*	>38 h	Yes	1.74	25 min	No	8
aptamer–CQDs and GNP	*S. aureus*	>26 h	Yes	10	90 min		9
PDANSs-FAM-Apt	*S. aureus*	>24.5 h	Yes	1	6 h	No	10
CsWO3–FCD naNohybrid	*E. coli* and *S. aureus*	>71 h	Yes	70 and 131	>25 h	No	11
CRISPR-Cas12a-Assisted MXene	*S. aureus*	45 min	Yes	23	2 h	No	12
(Apt-MBs)-(FcMBL@AuNCs)	MRSA and KPC-2 KP[b]	>73 h	Yes	20	15 min	Yes	13
Van-MBs@S. aureus@biotin-pig IgG@SA@biotin-RCA	*S. aureus*	>6h	Yes	3.3×10^2	113 min	Yes	14
Ab@SPM	*E. coli*	>12 h	Yes	2	55 min	No	15
Fe_3O_4@aptamer	*Listeria monocytogenes*	11h	Yes	1.0×10^2	20 min	Yes	16
Zr-mMOF-p-Ab-Apt- F@UIO-66-NH2-p-LPS-Apt	*A. baumannii*	>12 h	Yes	10	2.5 h	Yes	17
APBA-PEG-MNs	*S. aureus*	6 h	Yes	2.7×10^2	80 min	Yes	18
Paper@epoxy@PEI-DFFPBA	*S. aureus*	>88 h	Yes	2.24×10^2	30 min	Yes	19

MNP-APTs@QDs-Abs	*Alicyclobacillus acidoterrestris*	>21 h	Yes	10^3	90 min	Yes	20
Eu^{3+}	*S. aureus, E. coli, Enterococcus faecalis, A. baumannii Bacillus cereus,* and *P. aeruginosa*	2.25 min	No	10^4	~1 h	Yes	This work

[a]Ab: antibody; Abs: antibody; APBA: 3-amiNophenylboronic acid; Apt: aptamer; AuNC: gold naNocluster; AuNPs: gold naNoparticles; BCD: blue carbon dots; BSA: bovine serum albumin; CQDs: carbon quantum dots; CRISPR: clustered regularly interspaced short palindromic repeats; DFFPBA: 3,5-difluoro-4-formylphenylboronic acid; FAM: fluorophore 6-carboxyfluorescein-terminated S. aureus; FCD: fluorescent carbon dots; FcMBL: fragment crystallizable manNose binding lectin; FCyP: cyaNostyryl-furan and pyridinium salt; FL: fluorescein; GNP: gold naNoparticles; GO: graphene oxide; LPS: lipopolysaccharide; LUM: lumiNol; MBs: magnetic beads; mMOF: magnetic based metal organic framework; MNP: magnetic naNoparticles; MNs: magnetic naNomaterials; MRSA: methicillin-resistant *Staphylococcus aureus*; MTPE: methoxy modified tetraphenylethylene; NCND: nitrogen-doped carbon naNodots; o-CD: ortho-phenylenediamines carbon dot); PDANSs: polydopamine naNospheres; PEG: polyethylene glycol; PEI: polyethyleneimine; QDs: quantum dots; RCA: rolling circle amplification; SA: streptavidin; SPM: superparamagnetic magnetic beads; Van: vancomycin; ZIF: zeolitic imidazolate framework. [b]KPC-2 KP: *Klebsiella pneumoniae* carbapenemase 2-expressing *Klebsiella pneumoniae*

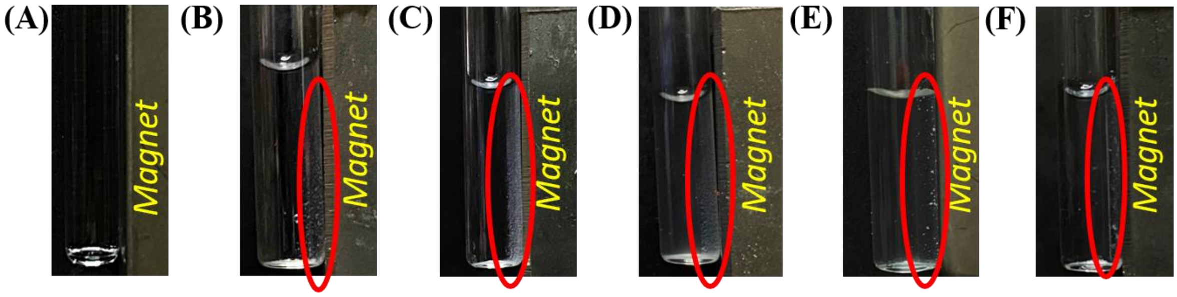

Figure S1. Examination of the magnetic Eu^{3+}-bacterium conjugates. Photographs of the (A) blank sample (0.39 mL) containing Tris buffer at pH 8 only and the bacterial samples (0.39 mL, 0.2 mg mL^{-1}) including (B) *P. aeruginosa*, (C) *E. faecalis*, (D) *B. cereus*, (E) *E. coli* J96, and (F) *A. baumannii* prepared in Tris buffer (pH 8) obtained with the addition of Eu^{3+} (75 mM, 10 μL) followed by microwave-heating (power: 180 W) for 2.25 min and magnetic isolation by placing an external neodymium magnet (~4000 G). The photographs were taken under room light. The red oval indicates the location of the magnetic Eu^{3+}-bacterium conjugates.

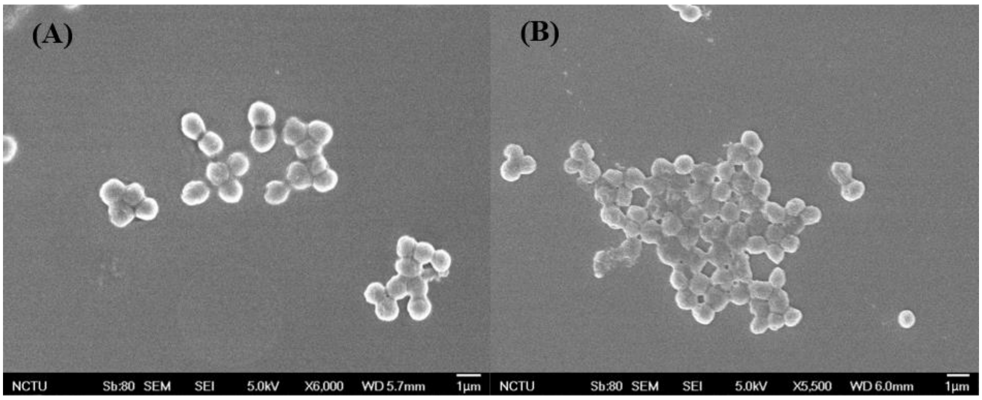

Figure S2. SEM images of *S. aureus* obtained (A) before and (B) after microwave-heating (power: 180 W) for 2.25 min.

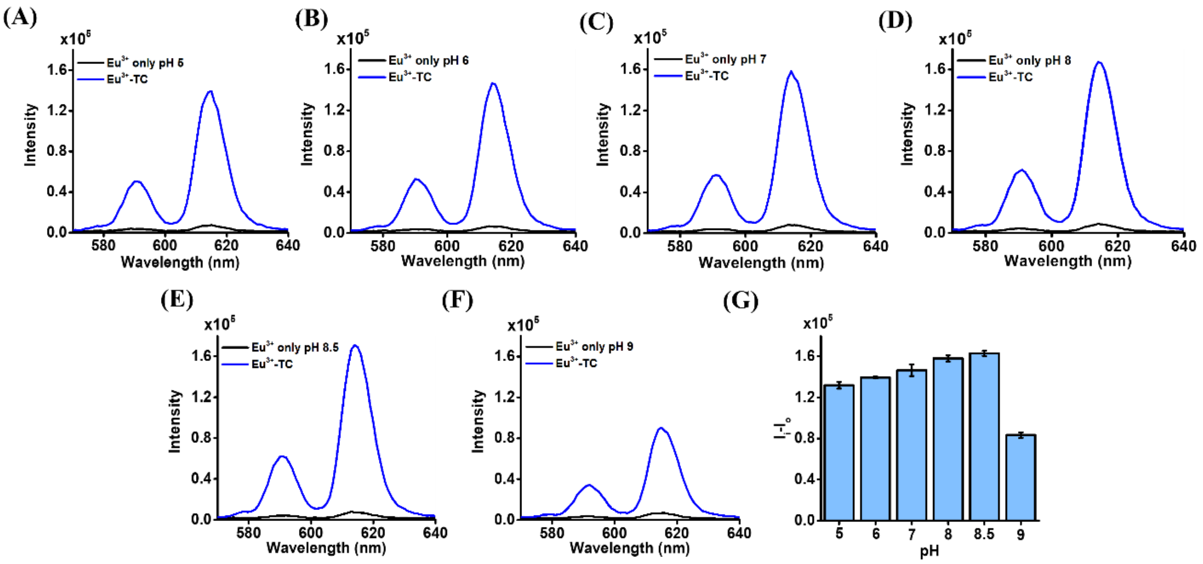

Figure S3. Examination of the optimal pH for enhancing fluorescence intensity derived from Eu^{3+} by TC. Representative fluorescence spectra (λ_{ex}= 394 nm) of the samples containing Eu^{3+} (2 mM) obtained without (black) and with (blue) adding TC (13 μM) at pH (A) 5, (B) 6, (C) 7, (D) 8, (E) 8.5, and (F) 9 after vortex-mixing for 1 h. Three replicates were conducted. (G) Bar graphs of the summarized results derived from Panels (A)-(F). I_i and I_0 stand for the fluorescence intensity at 616 nm of the black and blue spectra, respectively.

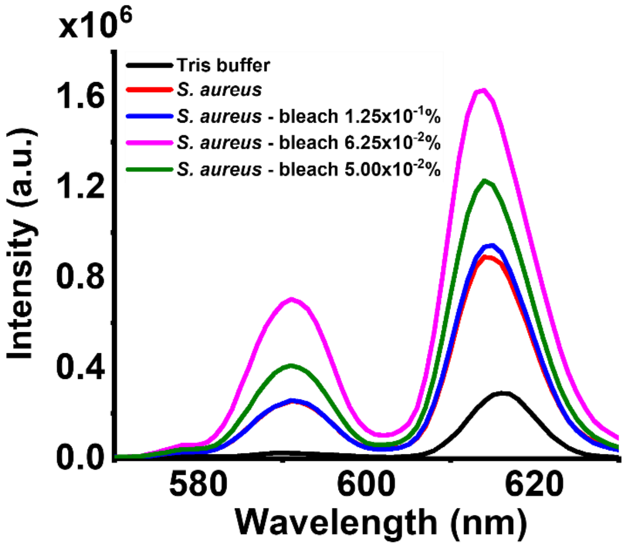

Figure S4. Optimization of the concentration of the added bleach. Fluorescence spectra (λ_{ex}= 394 nm) of the samples containing *S. aureus* (0.02 mg mL^{-1}, 0.39 mL) obtained by using Eu^{3+} (75 mM, 10 μL) as the trapping probe followed by magnetic isolation and the addition of bleach with different concentrations under microwave-heating (power: 180 W, 1.75 min) and added with TC (30 μM, 30 μL) incubated under microwave-heating (power: 180 W, 1.75 min).

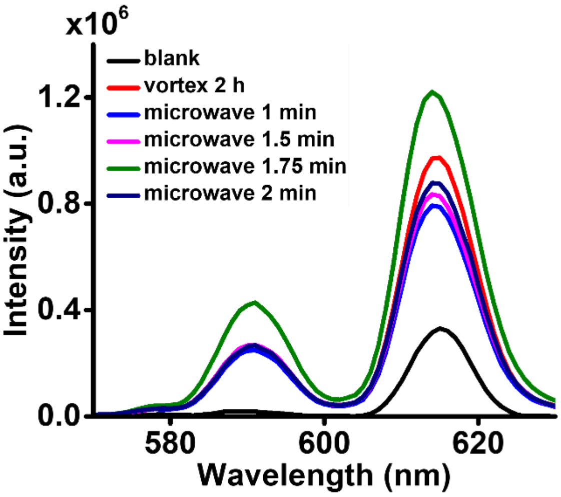

Figure S5. Examination of incubation conditions. Fluorescence spectra (λ_{ex}= 394 nm) of the samples (0.39 mL) containing *S. aureus* (0.02 mg mL^{-1}) obtained after trapped by Eu^{3+} (75 mM, 10 μL) followed by bleach treatment (6.25×10^{-2}%) and the addition of TC (30 μM, 30 μL) under different incubation conditions.

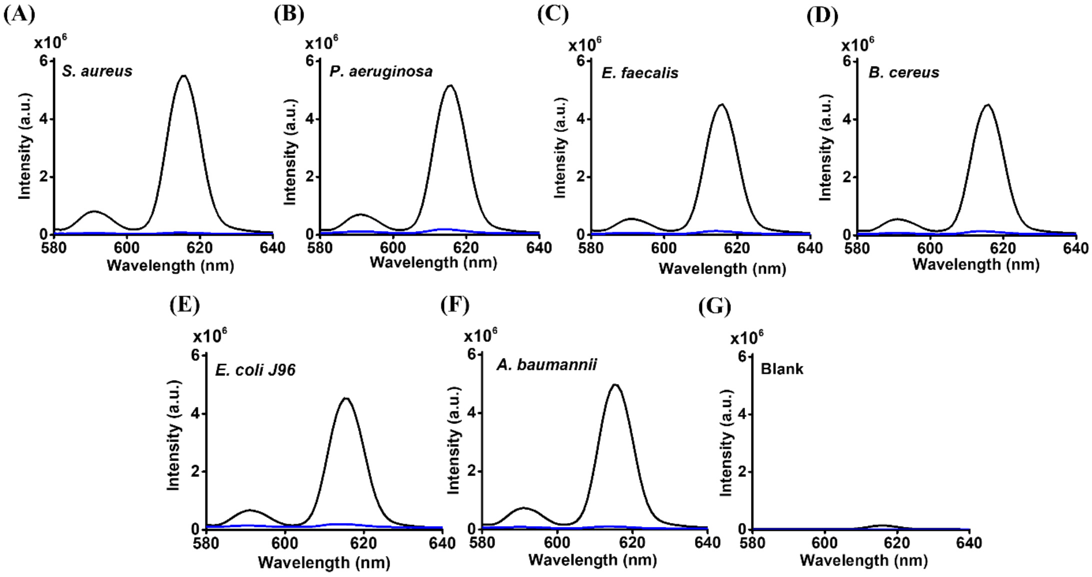

Figure S6. Examination of different model bacteria. Fluorescence spectra of the bacterial samples including (A) *S. aureus*, (B) *P. aeruginosa*, (C) *E. faecalis*, (D) *B. cereus*, (E) *E. coli* J96, (F) *A. baumannii*, and (G) blank obtained before (blue) and after (black) bleach treatment and the addition of TC. The concentrations of all the bacteria in the samples were 0.2 mg mL^{-1}. All the samples (0.39 mL) were added with Eu^{3+} (75 mM, 10 μL) followed by microwave-heating (power: 180 W) for 2.25 min and magnetic isolation by placing an external neodymium magnet (~4000 G) followed by rinse steps. Bleach (0.25%, 10 μL) was added to the individual samples with the final concentration of 6×10^{-3} % and incubated under microwave-heating (power: 180 W) for 1.75 min. TC (30 μM, 30 μL) was added to the individual samples and incubated under microwave-heating (power: 180 W) for 1.75 min in the water bath of 2 mL.

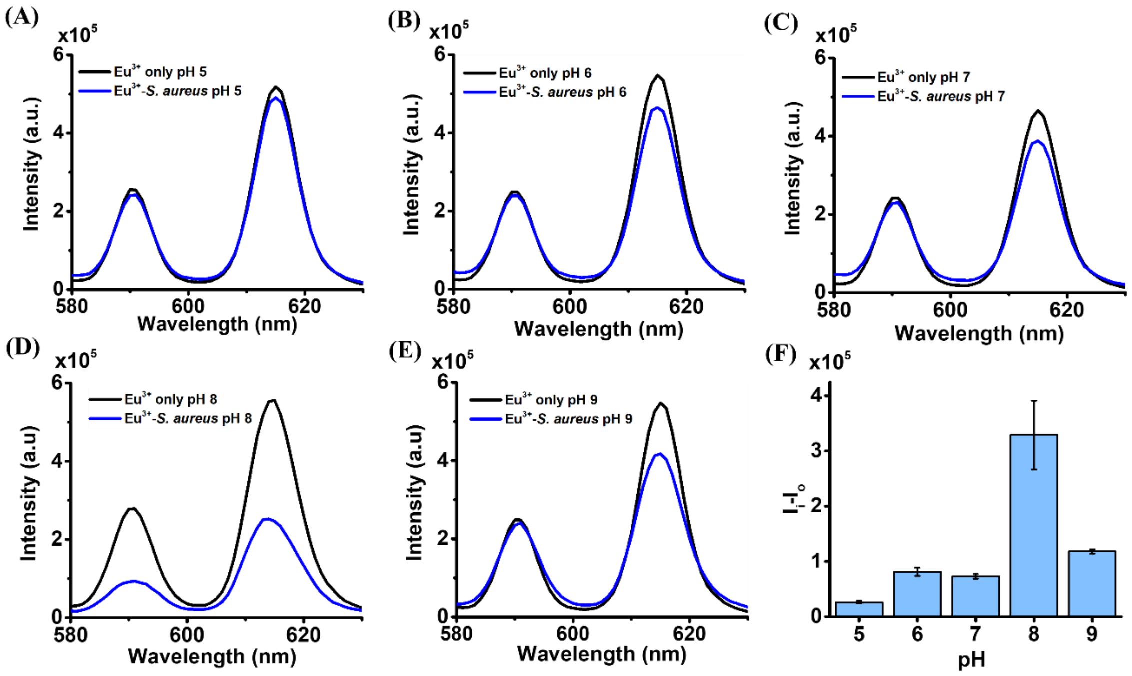

Figure S7. Examination of the optimal binding pH of Eu^{3+} toward *S. aureus.* Representative fluorescence spectra (λ_{ex}= 394 nm) of the supernatants derived from the samples (0.39 mL) containing Eu^{3+} (10 mM) obtained before and after incubated with *S. aureus* (0.2 mg mL^{-1}) at pH (A) 5, (B) 6, (C) 7, (D) 8, and (E) 9 followed by centrifugation (3000 rpm, 5 min). Three replicates were conducted for different pH conditions. (F) Bar graphs of the summarized results of Panels (A)-(E) obtained from different pH values (n= 3) in terms of binding capacity of Eu^{3+} onto *S. aureus*. I_i and I_0 stand for the fluorescence intensity at 616 nm of the black and blue spectra, respectively.

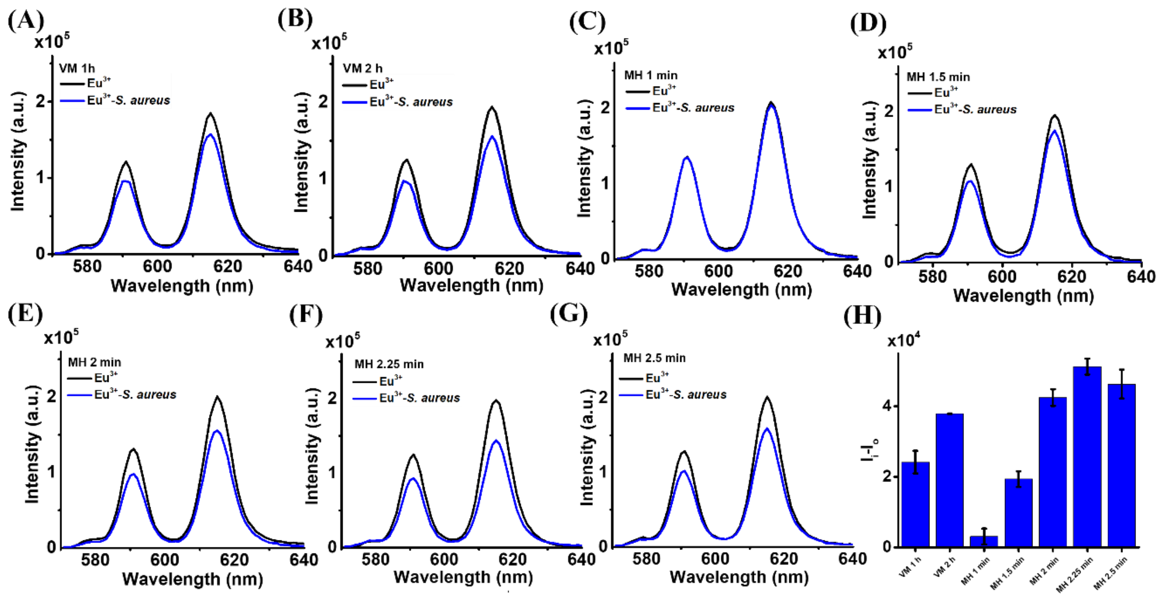

Figure S8. Examination of the optimal incubation parameters. Representative fluorescence spectra (λ_{ex}= 394 nm) of supernatants of the samples (0.39 mL) containing Eu^{3+} (2 mM) obtained without (black) and with (blue) incubated with *S. aureus* (0.2 mg mL^{-1}) under vortex-mixing (VM) for (A) 1 h, (B) 2 h, and microwave-heating (MH) (power: 180 W) for (C) 1 (D) 1.5, (E) 2, (F) 2.25, and (G) 2.5 min followed by magnetic isolation. Three replicates were conducted. (H) Bar graphs of the summarized results derived from Panels (A)-(G). I_i and I_0 stand for the fluorescence intensity at 616 nm derived from the black and blue spectra, respectively.

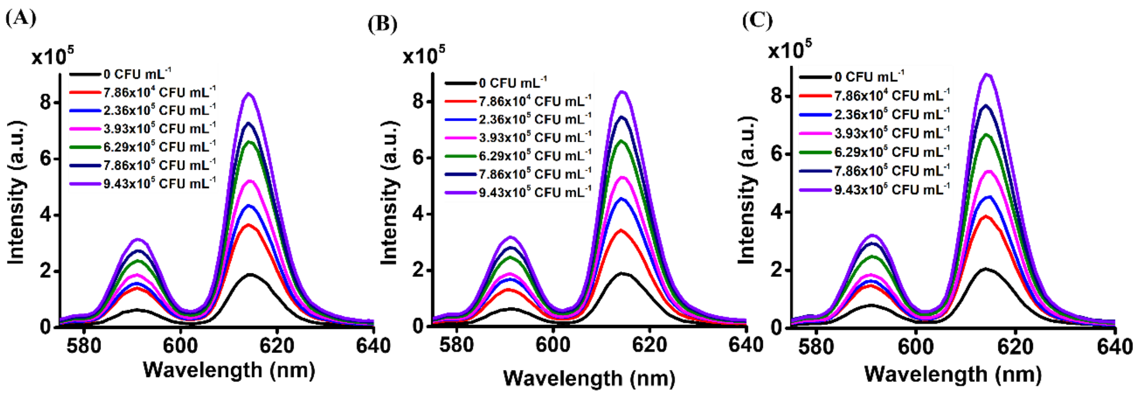

Figure S9. Quantitative analysis. Fluorescence spectra (λ_{ex}= 394 nm) of the samples (0.39 mL) containing *S. aureus* with different concentrations obtained by using Eu^{3+} (75 mM, 10 μL) as the trapping probe followed by rinse, bleach treatment (0.0625 %), and the addition of TC (30 μM, 30 μL). Three replicates (A)-(C) were conducted. *S. aureus* with the concentration of 1 mg mL^{-1} is equal to ~7.86×10^{9} CFU mL^{-1}.

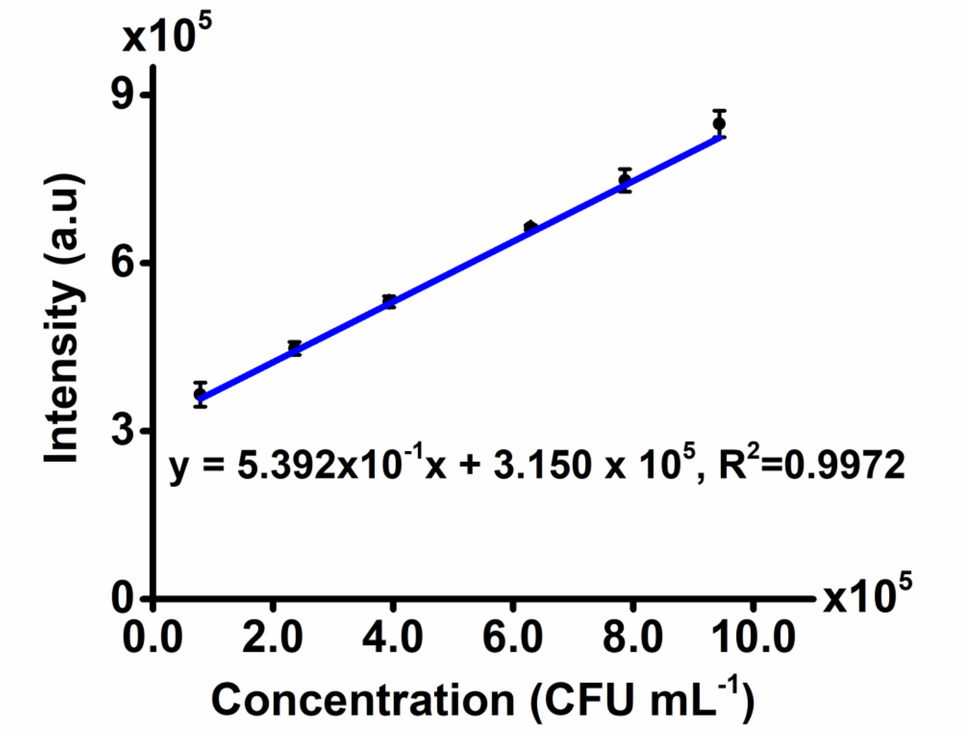

Figure S10. Calibration curve obtained by plotting the fluorescence intensity at the wavelength of 616 nm of the fluorescence spectra shown in Figure S9 versus the concentration of *S. aureus*.

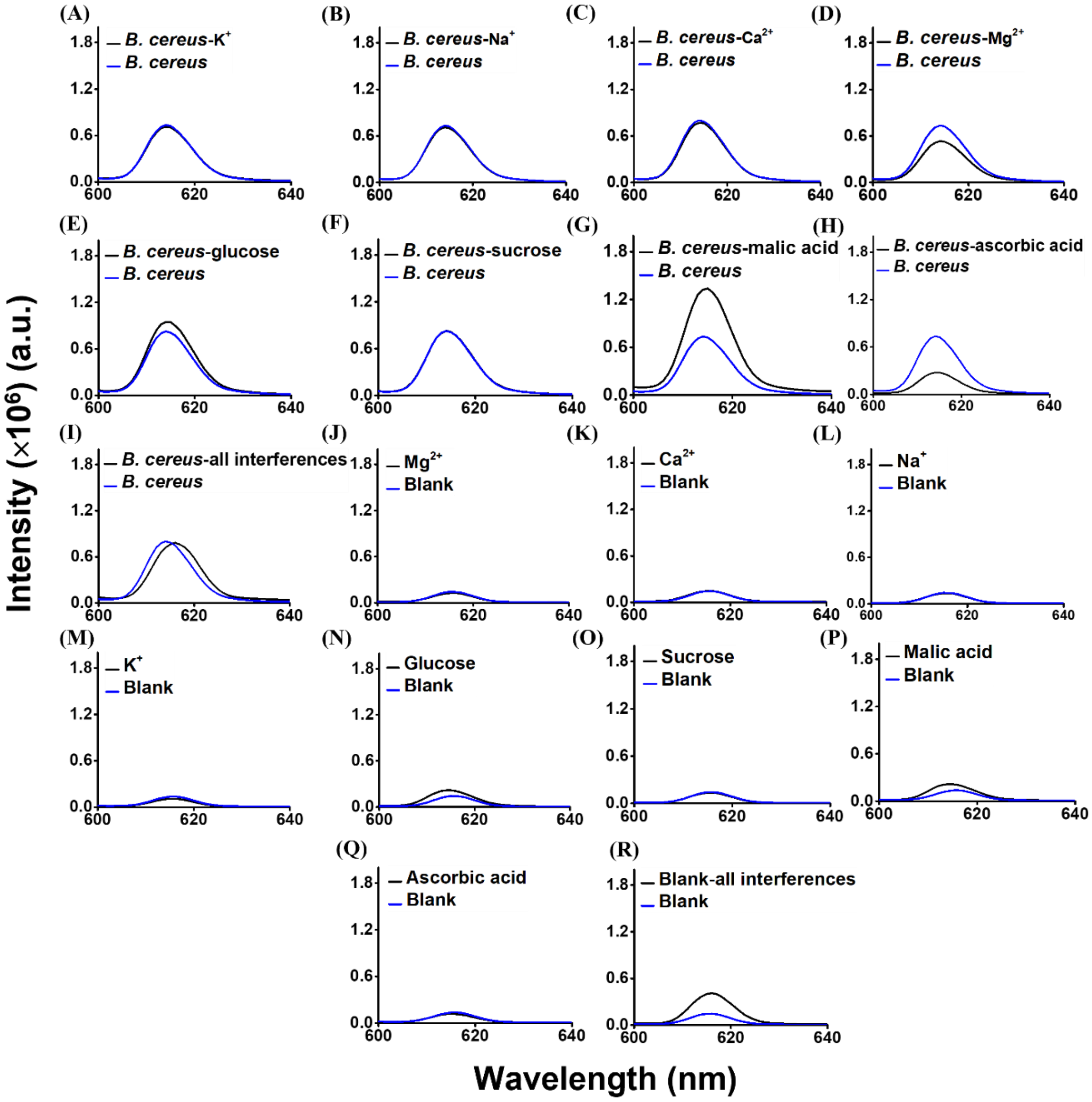

Figure S11. Fluorescence spectra (λ_{ex}= 394 nm) of (A) of the sample (390 μL) containing *B. cereus* (2 μg mL^{-1}) in the absence (blue) and presence (black) of the interference species including (A) Mg^{2+} (0.10 mg mL^{-1}, 390 μL), (B) Ca^{2+} (0.16 mg mL^{-1}, 390 μL), (C) Na^{+} (0.08 mg mL^{-1}, 390 μL), (D) K^{+} (2.02 mg mL^{-1}, 390 μL)), (E) glucose (52.60 mg mL^{-1}, 390 μL) ,(F) sucrose (25.20 mg mL^{-1}, 390 μL), (G) malic acid (0.90 mg mL^{-1}, 390 μL), and (H) ascorbic acid (0.02 mg mL^{-1}, 390 μL) (I) all interferences obtained after using our trapping and sensing method. Fluorescence spectra of the Tris buffer (10 mM, pH 8) without (blue) and with (black) the addition of the interference species including (J) Mg^{2+} (0.10 mg mL^{-1}, 390 μL), (K) Ca^{2+} (0.16 mg mL^{-1}, 390 μL), (L) Na^{+} (0.08 mg mL^{-1}, 390 μL), (M) K^{+} (2.02 mg mL^{-1}, 390 μL)), (N) glucose (52.6 mg mL^{-1}, 390 μL) ,(O) sucrose (25.2 mg mL^{-1}, 390 μL), (P) malic acid (0.90 mg mL^{-1}, 390 μL), (Q) ascorbic acid (0.02 mg mL^{-1}, 390 μL), and (R) all interferences above obtained after using our method.

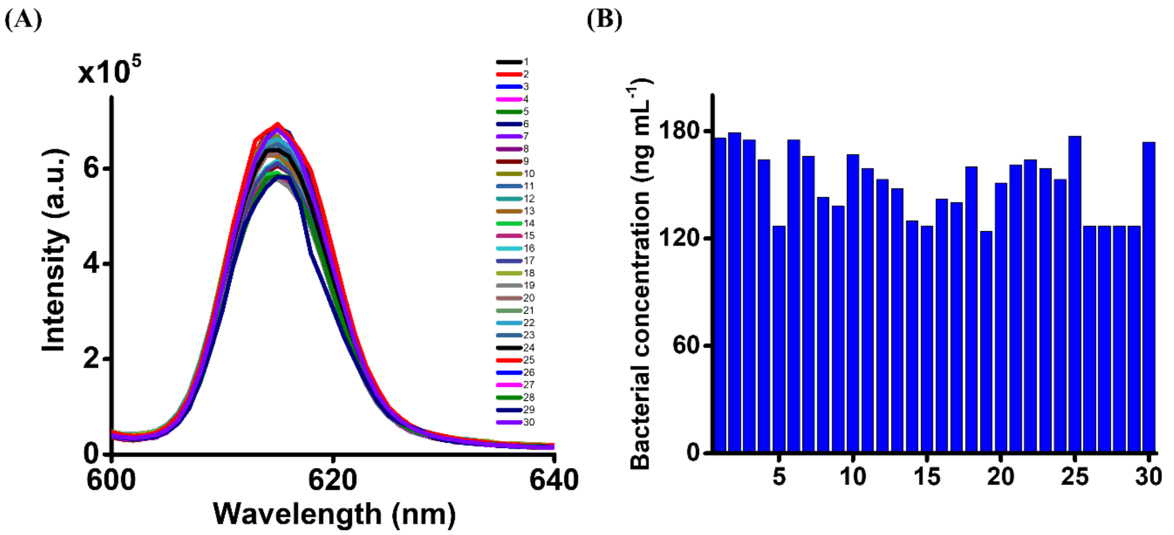

Figure S12. (A) Examination of precision and accuracy. (B) Fluorescence spectra of the sample containing *S. aureus* (150 ng mL^{-1}) obtained after treated by our developed method for 30 runs (6 runs per day for 5 days).

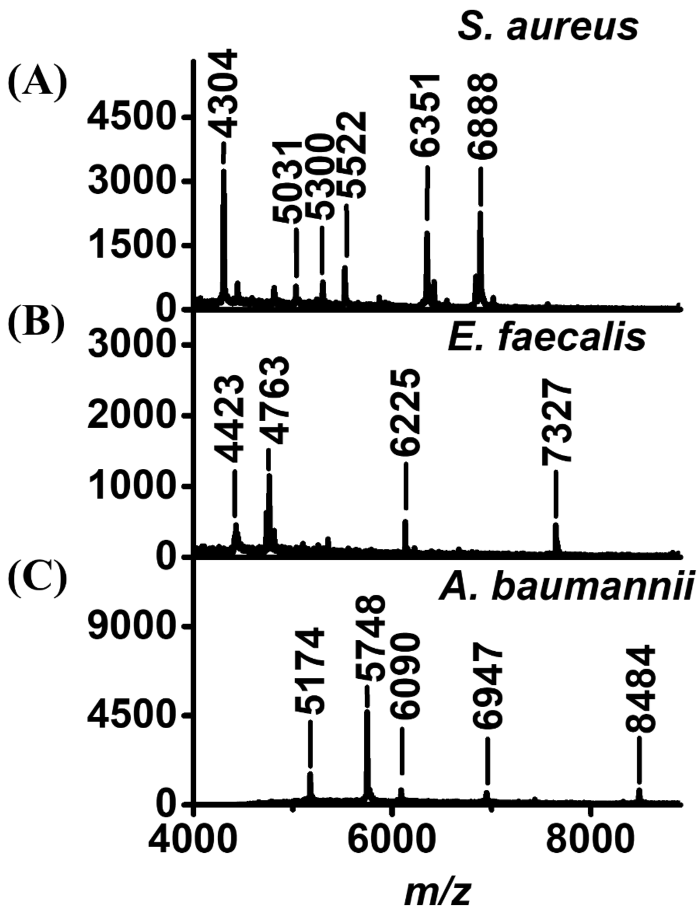

Figure S13. MALDI mass spectra of the samples (0.1 mg mL^{-1}) including (A) *S. aureus*, (B) *E. faecalis*, and (C) *A. baumannii*. CHCA (20 mg mL^{-1}) prepared in the solvent containing acetonitrile and 3% trifluoroacetic acid (2:1, v/v) was used as the MALDI matrix.

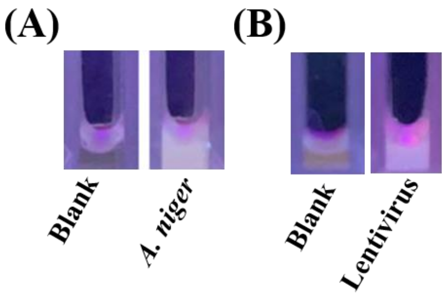

Figure S14. Examination of our method for sensing of other microorganisms including the samples (0.39 mL) containing (A) *A. niger* spores (OD_{600}= 0.2) and (B) lentiviruses (~5×10^7 RIU mL^{-1}).

Supporting Information time-lapse Video 1. Time-lapse video showing the monitoring of a Tris buffer (0.39 mL, pH 8) with the addition of Eu^{3+} (75 mM, 10 μL). The resulting solution was incubated in a microwave oven (power: 180 W) for 2.25 min before magnetic isolation with an external neodymium magnet (~4000 G) placed nearby.

Supporting Information time-lapse Video 2. Time-lapse video showing the monitoring of a sample solution (0.39 mL) containing *S. aureus* (0.5 mg mL^{-1}) prepared in Tris buffer (pH 8), with the addition of Eu^{3+} (75 mM, 10 μL). The sample was incubated in a microwave oven (power: 180 W) for 2.25 min before magnetic isolation with an external neodymium magnet (~4000 G) placed next to the sample tube.

References

1. Feng, Q.; Xu, H.; Li, Z.; Chen, C.; Miao, X. *Sens. Actuators B Chem.* **2022,** *372,* 132598.
2. Shen, Y.; Wu, T.; Zhang, Y.; Ling, N.; Zheng, L.; Zhang, S.-L.; Sun, Y.; Wang, X.; Ye, Y. *Anal. Chem.* **2020,** *92,* 13396−13404.
3. Bahari, D.; Babamiri, B.; Salimi, A.; Salimizand, H. *Talanta.* **2021,** *221,* 121619.
4. Li, J.; Fang, Y.; Lin, X.; Hao, Z.; Yin, Y.; Zhao, M.; Liu, Y. *ACS Appl. Bio Mater.* **2021,** *4,* 6361−6370.
5. Fu, L.; Chen, Q.; Jia, L. *Food Chem.* **2022,** *374,* 131750.
6. Balakrishna, R. G. *Sens. Actuators B Chem.* **2023,** *397,* 134626.
7. Fu, F.; Zhang, Y.; Li, L.; Wang, H.; Li, Q.; Tao, X.; Song, Y.; Song, E. *Anal. Chem.* **2020,** *92,* 11462-11468.
8. Wu, H.; Fang, Y.; Tian, L.; Liu, X.; Zhou, X.; Chen, X.; Gao, H.; Qin, H.; Liu, Y. *ACS Sens.* **2023,** *8,* 3205−3214.
9. Pebdeni, A. B.; Hosseini, M.; Ganjali, M. R. *Food Anal. Methods.* **2020,** *13,* 2070-2079.
10. Ye, Y.; Zheng, L.; Wu, T.; Ding, X.; Chen, F.; Yuan, Y.; Fan, G.-C.; Shen, Y. *ACS Appl. Mater. Interfaces.* **2020,** *12 (31),* 35626-35637.
11. Robby, A. I.; Kim, S. G.; Lee, U. H.; In, I.; Lee, G.; Park, S. Y. *Chem. Eng. J.* **2021,** *403,* 126351.
12. Sheng, A.; Wang, P.; Yang, J.; Tang, L.; Chen, F.; Zhang, J. *Anal. Chem.* **2021,** *93,* 4676-4681.
13. Sun, J.; Zhang, L.; Xu, Y.; Xue, Y.; Qiao, L.; Ding, C.; Ling, L.; Yu, S. *Sens. Actuators B Chem.* **2021,** *349,* 130762.
14. Wang, Y.; Wang, Z.; Zhan, Z.; Liu, J.; Deng, T.; Xu, H. *Anal. Chim. Acta.* **2022,** *1189,* 339213.
15. Rodoplu, D.; Chang, C.-S.; Kao, C.-Y.; Hsu, C.-H. *Talanta.* **2021,** *230,* 122291.

16. Du, J.; Liu, J.; Liu, K.; Zhao, D.; Sagratini, G.; Tao, J.; Bai, Y. *Anal. Methods.* **2022,** 14, 2188-2194.

17. Yang, S.; Guo, Y.; Fan, J.; Yang, Y.; Zuo, C.; Bai, S.; Sheng, S.; Li, J.; Xie, G. *Mikrochim. Acta.* **2020,** *187,* 1-10.

18. Wang, Z.; Feng, X.; Xiao, F.; Bai, X.; Xu, Q.; Xu, H. *Microchem. J.* **2022,** *178*, 107379.

19. Xu, Y.; Zheng, H.; Sui, J.; Lin, H.; Cao, L. *Foods.* **2023,** *12 (7),* 1366.

20. Tian, Y.; Li, X.; Cai, R.; Yang, K.; Gao, Z.; Yuan, Y.; Yue, T.; Wang, Z. *Food Control.* **2021,** *126*, 108060.
